# Estimating weaning duration from incremental dentine δ^15^N and δ^13^C using a sequence-based LSTM neural network: A deep learning framework for bioarchaeological applications

**DOI:** 10.1371/journal.pone.0337619

**Published:** 2025-12-17

**Authors:** Elissavet Ganiatsou, Angelos Souleles, Christina Papageorgopoulou

**Affiliations:** 1 Laboratory of Biological Anthropology, Department of Humanities, Democritus University of Thrace, Komotini, Greece; 2 Institute of Applied Bioarchaeological Research, Democritus University of Thrace, Komotini, Greece; Vilnius University: Vilniaus Universitetas, LITHUANIA

## Abstract

The estimation of weaning duration from incremental dentine δ^15^N and δ^13^C values offers insights into health, nutrition, and demography in past populations. In this study, we developed a novel machine learning approach to estimate weaning duration using a sequence-based Long Short-Term Memory (LSTM) neural network model. The model was trained on published serial isotopic data from 279 individuals across three tooth types (M1, dM1, dM2). We enhanced predictive performance by incorporating temporal features (differences, rolling averages, slopes) and encoding tooth type to allow the model to learn type-specific patterns. The model was evaluated with RMSE, MAE, and R^2^ metrics using Monte Carlo dropout to quantify predictive uncertainty. As a proof of concept, we compared our model to established methods in weaning estimation (WEAN, ChangeR) using 20 individuals from the site of Thessaloniki. Our model achieved an RMSE of 0.46 years (95% CI: 0.38–0.54), a MAE of 0.36 years (95% CI: 0.29–0.44), and an R^2^ of 0.82 (95% CI: 0.73–0.88), indicating reliable predictive accuracy. In contrast to previous approaches that rely primarily on δ^15^N, our method leverages sequential patterns in both δ^15^N and δ^13^C to infer the weaning process. This study introduces the first machine learning framework specifically designed to estimate weaning duration from serial isotopic data. By modelling complex, non-linear developmental trajectories, it offers a scalable and robust tool for reconstructing weaning practices in bioarchaeological and related research studies.

## Introduction

Weaning, the gradual cessation of breastfeeding accompanied by the introduction of complementary foods, represents a pivotal developmental transition in the human life course [1–3]. It may be conceptualized either as a discrete event (the complete cessation of breastfeeding or weaning age) or, more commonly, as a protracted process involving the progressive substitution of breast milk with non-maternal dietary sources [4]. Understanding the timing and duration of weaning has far-reaching implications for health, nutritional status, and cognitive development [5–7]. While weaning is a biologically universal process among mammals, its onset and duration in humans are highly variable, influenced by a cultural, environmental, and economic factors [1,2,8–10]. In archaeological contexts, reconstructing weaning strategies is critical for elucidating demographic patterns, maternal investment, and the epidemiology of childhood morbidity and mortality [11].

The investigation of weaning practices past populations is based on biomolecular data from skeletal and dental tissues [11]. Among the most robust biochemical methods for reconstructing early-life feeding practices is the analysis of nitrogen stable isotope ratios (δ^15^N) in incremental dentinal collagen [12]. This analysis offers the opportunity to reconstruct individual isotopic profiles that preserve a high-resolution record (6 months or less) of dietary inputs during childhood. The core assumption to detect breastfeeding is that, exclusively breastfed infants typically exhibit elevated δ^15^N values compared to their mothers, attributable to trophic level enrichment, and a gradual decline in δ^15^N across successive dentinal sections, which reflects the transition through weaning [13–15]. This method has gained considerable traction in recent years, with more than 5000 individual δ^15^N measurements reported in Europe alone [16]. This underscores the growing interest in reconstructing infant feeding trajectories at the individual level.

Over the past five years, there has been a surge of interest in developing statistical and mathematical approaches to estimate weaning duration more systematically. Ganiatsou, Souleles, and Papageorgopoulou [17] introduced the software WEAN, which applies a straightforward mathematical method to identify the point of δ^15^N decline (elbow). Similarly, Velte et al. [18] proposed an approach that identifies the weaning completion age by comparing the sequence of the δ^15^N and reporting weaning cessation as an age range, offering a more realistic interpretation of the gradual nature of weaning [19]. More recently, Cocozza et al. [20] introduced the ChangeR algorithm, which detects changepoints in δ^15^N values using the R package *mcp* and incorporates prior information to model temporal trends.

While these methods are particularly useful and efficient, they are not without limitations. They mostly rely on simplified curve-fitting techniques or require a priori assumptions regarding the shape and trend of inflection points along the isotopic curve. Although human dentition has a robust developmental timeframe, empirical evidence exists that dentine apposition and growth vary among populations [21]. These constraints limit the adaptability, scalability, and precision of weaning reconstructions across diverse archaeological populations. As a consequence, there are inherent limitations in current methods of weaning duration estimation, which represent a critical methodological gap in bioarchaeological research.

The framework of Machine Learning (ML) includes exceptional approaches for such a task. Currently, no dedicated machine learning model exists to estimate the full trajectory or duration of weaning from δ^15^N and δ^13^C sequences. The development of such a tool has the potential to surpass current methods as ML has the capacity to model complex, non-linear, and individualized patterns.

In response to this gap, the present study introduces a novel supervised deep learning model using a sequence-based Long Short-Term Memory (LSTM) architecture to estimate the duration of weaning in archaeological populations based on δ^15^N and δ^13^C data of incrementally sampled dentinal collagen. In contrast to other statistical approaches, this model applies supervised learning to empirical datasets, allowing it to detect latent, non-linear structures in isotopic trajectories that reflect individual variation in weaning behavior. Given the central role of weaning studies in understanding paleodemography, early-life health, and human life history evolution, the development of a replicable, scalable, and high-resolution computational tool is both timely and imperative. This research offers a significant methodological advancement in reconstructing early-life dietary practices from biomolecular data through the integration of bioarchaeological science with advanced machine learning techniques.

## Materials and methods

### Model development

#### Data collection and preprocessing.

**Fig 1:**
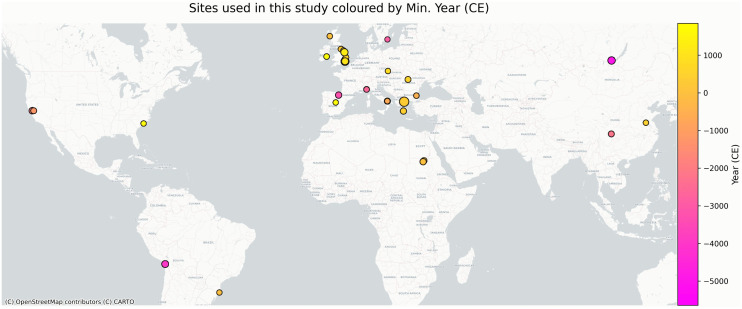
Geographic distribution of archaeological sites included in this study. Each point represents a site, with marker size proportional to the number of samples analyzed. Points are color-coded by the minimum date of the samples (CE), with the color gradient shown in the accompanying colorbar. Base map generated from Natural Earth public domain data (naturalearthdata.com).

We compiled a dataset comprising published δ^15^N and δ^13^C measurements (2868) from 279 individuals (Table 1). We have sampled individuals almost across all continents spanning from 5644 BCE to 1845 CE. Fig 1 shows the temporal and spatial variation of the individuals included in this study. Individuals were included in the analysis only if they had sufficiently complete isotopic sequences to capture temporal patterns. Specifically, we excluded individuals with fewer than three measurements or with extensive missing age-at-increment data, as these incomplete profiles could obscure trends and reduce model performance. S1 Fig shows the distribution of isotopic measurements for each tooth type. The resulting dataset consisted of five key variables: sample_id, age_at_formation, δ^15^N, δ^13^C and tooth_type. The sample_id denotes the unique identifier assigned to each individual, while age_at_increment represents the median age associated with each δ^15^N and δ^13^C value. The tooth_type variable indicates the anatomical classification of each sampled tooth (S1 Table).

**Table 1:** Summary of the dataset used in this study.

Tooth type	Number of individuals	Total number of measurements
M1	227	2479
dM1	23	179
dM2	29	208
Total	279	2868

We focused on three tooth types commonly employed in reconstructing breastfeeding and weaning behaviors [19]: first permanent molars (M1), deciduous first molars (dM1), deciduous second molars (dM2). The raw dataset was sorted by sample_id and age_at_formation. To ensure consistency across features and facilitate model convergence, we normalised the variables δ^15^N, δ^13^C, and age-at-increment using StandardScaler (z-score normalization).

The table includes the total number of δ^15^N and δ^13^C measurements per individual and the number of individuals analyzed for each tooth type (M1: first permanent molars, dM1: deciduous first molar, dM2: deciduous second molar)

### Feature Engineering

Several temporal and derivative features were computed to improve model learning. Specifically:

- differences (Δ) between consecutive measurements based on age-at-increment within each individual for δ^15^N and δ^13^C were calculated to capture the local direction and magnitude of isotopic change, which reflect the pace and progression of dietary transition.
- a centered 3-point rolling mean was computed for δ^15^N and δ^13^C to smooth short-term variability and highlight underlying biological trends in isotopic composition, minimizing the influence of analytical noise.
- local linear regression (window size = 3) was applied to δ^15^N and δ^13^C to estimate the instantaneous rate of isotopic change over time, providing an explicit measure of how rapidly dietary signals shifted during tooth formation (slope).

These features, along with age and original δ^15^N and δ^13^C values, were used as input for the model.

### Weaning estimation

We estimated individual weaning ages following the logic implemented in the WEAN software. Specifically, by identifying the “elbow” of δ^15^N values without applying regression. This approach was designed to emulate the traditional “eye-balling” method but did not perform regression to avoid overfitting. Although our model incorporated δ^13^C values, the initial weaning estimation relied exclusively on δ^15^N, which current scholarship considers the more robust indicator of breastfeeding and weaning [14,18,22]. Estimated weaning durations were then normalized globally using RobustScaler, which uses the media and inter-quantile range (IQR), to reduce sensitivity to outliers and ensure comparability across individuals.

### Sequence preparation

For each individual, time series-sequences were constructed using all engineered features: δ^15^N, δ^13^C, age-at-formation, Δ features, rolling averages and slopes. Because individuals differed in the number and spacing of their isotopic measurements, we implemented several pre-processing steps to standardize sequences. First, all isotopic values were ordered by age-at-formation. Missing intermediate values were left as gaps rather than imputed, and sequences of unequal length were padded with zeros to a common maximum length. In the neural network, these padded values were later masked, ensuring that they did not contribute to model training. These procedures allowed the model to learn time-dependent patterns while remaining robust to variation in sampling density.

To account for potential differences in isotopic patterns among tooth types, the categorical variable "tooth type" was one-hot encoded, i.e., converted into a binary vector representing each class (M1 = [1, 0, 0], dM1 = [0, 1, 0], dM2 = [0, 0, 1]). This encoding was repeated across all time steps in each individual’s isotopic sequence so that the model received explicit information about tooth type at every measurement. By providing this categorical context, the neural network could distinguish isotopic patterns driven by developmental timing rather than treating all teeth as a single, homogeneous group.

### Model architecture

At the core of the model is a Bidirectional Long Short-Term Memory (Bi-LSTM) layer. LSTM networks are a type of recurrent neural network that excels in learning and retaining long-term dependencies in time-series data. In the context of this model, the Bi-LSTM processes the dentine trajectories in both forward and backward directions. This bidirectional processing allows the model to capture information not only from past time steps but also from future time steps. As a result, the model is able to consider the entire context of the sequence when making weaning duration predictions, rather than being limited to just past observations. We chose this structure given that our δ^15^N and δ^13^C sequences represent complete tooth formation records ordered from earliest to latest dentine increments. The architecture consisted of a masking layer to ignore padded values, two stacked bidirectional LSTM layers with dropout regularization to prevent overfitting and a final dense layer with a single output predicting normalized weaning duration.

### Training

The data were segmented into training and test sets using an 80−20 split at the individual level, ensuring that the sequences are properly distributed across the two sets. The model was then trained for up to 200 epochs, and the loss curves for both the training and validation sets were saved for visualization. The model was compiled using the Adam optimizer with a learning rate of 0.0005 and trained using Mean Squared Error (MSE) loss function, which calculates the squared average of the errors to quantify the difference between the predicted and actual values. To prevent overfitting, an early stopping callback for 50 epochs was implemented. This callback halts the training process if the validation loss does not show improvement for 50 consecutive epochs, ensuring that the model does not continue to learn irrelevant patterns from the training data. Hyperparameters including the LSTM units, dropout rate, learning rate, and batch size were optimized through grid search, selecting the configuration with the lowest validation Root Mean Squared Error (RMSE).

### Uncertainty estimation

We used Monte Carlo Dropout (MC Dropout) at prediction time to quantify model uncertainty. The model was run 100 times with dropout active, generating a distribution of predictions for each test sample. Mean and standard deviation of these predictions were reported as the final estimate and uncertainty, respectively.

### Model evaluation

To evaluate the model’s performance, we calculated the RMSE, a widely used metric that measures the average magnitude of the errors between the predicted and actual values. This metric places a higher penalty on larger errors, making it sensitive to outliers. As such, RMSE provides an effective measure of the model’s overall prediction accuracy, especially when large deviations from the true values are detected. Moreover, we calculated the Mean Absolute Error (MAE), which measures the average absolute difference between the predicted and actual values, treating all errors equally regardless of their direction (i.e., positive or negative). Unlike RMSE, MAE does not disproportionately penalize large errors, making it a useful metric when the magnitude of error is of primary interest. Finally, we calculated the R-squared (R^2^), which quantifies the proportion of variance in the dependent variable (weaning duration) that is explained by the independent variables in the model. An R^2^ value closer to 1 indicates that the model’s predictions are highly aligned with the true values, reflecting a good fit. Conversely, a value closer to 0 suggests that the model’s predictions are less accurate and do not adequately explain the observed variability in the data. Bootstrap resampling (1000 iterations) was used to compute 95% confidence intervals for all metrics. Residuals were analyzed against maximum age-at-formation and tooth type to check for systematic biases. Individual-level prediction plots were generated showing δ^15^N and δ^13^C profiles, true and predicted weaning ages, and uncertainty bands.

All analyses were performed in Python 3.13.3, using TensorFlow 2.20.0 for neural network modeling, scikit-learn (version 1.6.1) for preprocessing and metrics, and matplotlib (version 3.10.1) and seaborn (version 0.13.2) for visualization. Models, scalers, and results were saved for reproducibility.

For using this approach on new datasets, the trained models have been integrated an executable application (currently available for Windows systems) with the intent to enable future analyses involving small sample sizes, such as cases with only one or two individuals, that may not be amenable to standard machine learning approaches. To promote transparency and reproducibility, the complete working code is freely available on the authors’ GitHub (see Code Availability Statement). This resource provides a robust framework for researchers seeking to train similar models on expanded or augmented datasets beyond those currently included.

### Model validation

To evaluate the performance of our model, we conducted a validation using 20 individuals from the Thessaloniki site [23] who had previously been analyzed with two alternative weaning estimation methods, WEAN [24] and ChangeR [20]. For each individual, δ^15^N and δ^13^C trajectories were plotted against age, and the weaning estimates from our model (LSTM), WEAN, and the ChangeR (95% Max CI) were overlaid to provide a visual assessment of agreement among the methods. Although the true weaning ages are unknown, this comparison allowed us to examine how our predictions align with established approaches.

We applied the Friedman test, a non-parametric repeated-measures ANOVA, to determine whether significant differences existed between WEAN, LSTM, and ChangeR estimates across samples. To evaluate the agreement between each pair of methods, we computed the Pairwise Concordance Correlation Coefficient (CCC), which measures both precision and accuracy between two sets of measurements. Pearson correlation coefficients were calculated to quantify linear relationships between the weaning estimates of each method. Correlation matrices were visualized using heatmaps for interpretability. For each pair of methods, Bland–Altman plots were constructed to examine systematic bias and limits of agreement. In these plots, the difference between two methods is plotted against their average for each sample, with ±1.96 standard deviations indicated to identify the range of typical differences [25].

## Results

### Weaning age predictions and evaluation of the model

**Fig 2:**
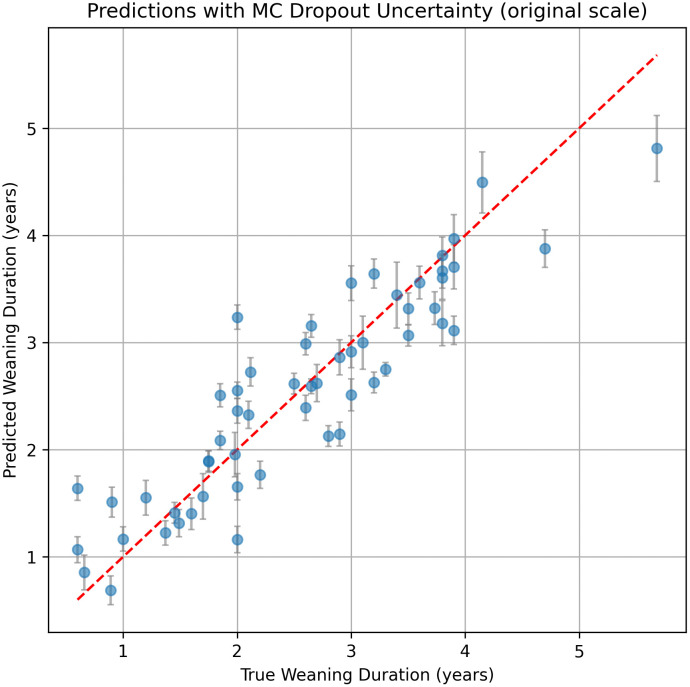
Scatterplot showing the alignment of weaning estimate predictions with the model of this study against their given original estimate.

**Fig 3:**
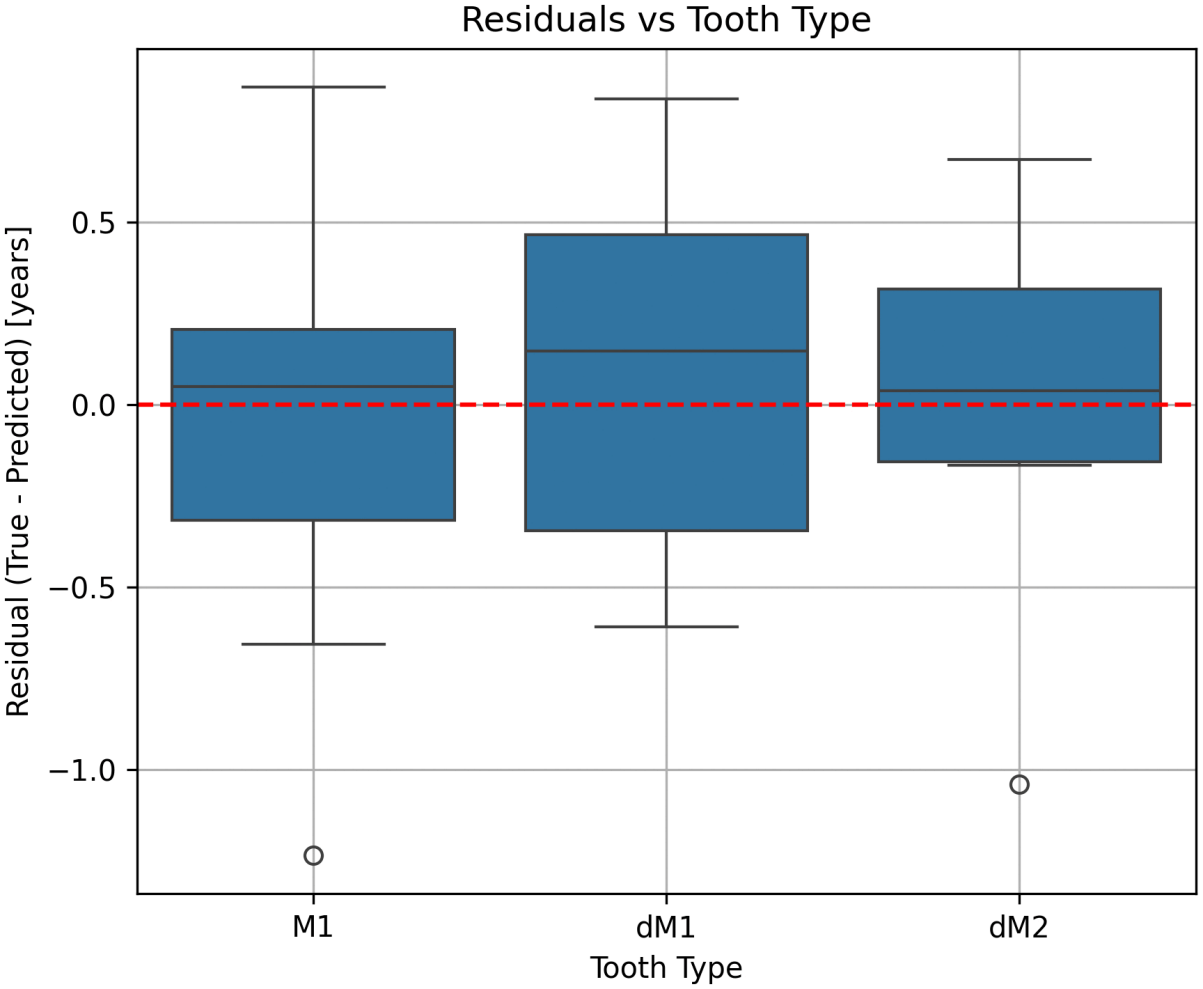
Residuals of predicted versus true weaning duration across tooth types. Box plots show the distribution of residuals (True − Predicted, in years) for M1, dM1, and dM2. The red dashed line represents zero residual, indicating perfect prediction.

After running the grid search, the best hyperparameters based on the R^2^ were LSTM units = 128, dropout rate = 0.4, learning rate = 0.001 and batch size = 8. The results of the model’s performance are summarized in Table 2 and Fig 2 and across tooth types in Fig 3. The RMSE was 0.46 years (95% CI: 0.38–0.54), and the MAE was 0.36 years (95% CI: 0.29–0.44), indicating that the predictions were, on average, within less than half a year of the observed weaning ages. The coefficient of determination was R^2^ = 0.82 (95% CI: 0.73–0.88), suggesting that the model explains a substantial portion of the variance in the weaning ages (Fig 2).

**Table 2:** Evaluation metrics for the LSTM model for weaning prediction.

RMSE	95% CI RMSE	MAE	95% CI MAE	R²	95% CI R²
0.46	0.38- 0.54	0.36	0.29- 0.44	0.83	0.73-0.88

### Predicted against true weaning ages

Fig 3 summarizes the distribution of model residuals, which represent the difference between the true and the predicted value (in years) for each analysed tooth type (M1, dM1, dM2). The horizontal red dashed line at y = 0 indicates unbiased prediction. Positive residuals reflect under-prediction, which means that prediction was lower than the true value, and negative residuals indicate over-prediction, which means that prediction was higher than the true value. Across all tooth types, residuals are centered close to zero, indicating overall unbiased model performance. However, subtle systematic differences are apparent. M1 samples show a median residual slightly above zero, suggesting a minor tendency toward under-prediction, with a moderate spread and a few negative outliers exceeding −1 year. dM1 exhibits the most pronounced bias, with the median residual clearly positive, indicating consistent underestimation of weaning duration. In contrast, dM2 displays a median slightly below zero, implying mild over-prediction, along with a modest negative skew and one or more extreme negative outliers. Despite these small location shifts, residual dispersion is comparable across tooth types, suggesting similar predictive uncertainty and variance structure in all subgroups. Outliers are infrequent but tend to occur on the negative side, reflecting occasional instances where the model over-predicted weaning duration (Fig 3).

### Learning curves

**Fig 4:**
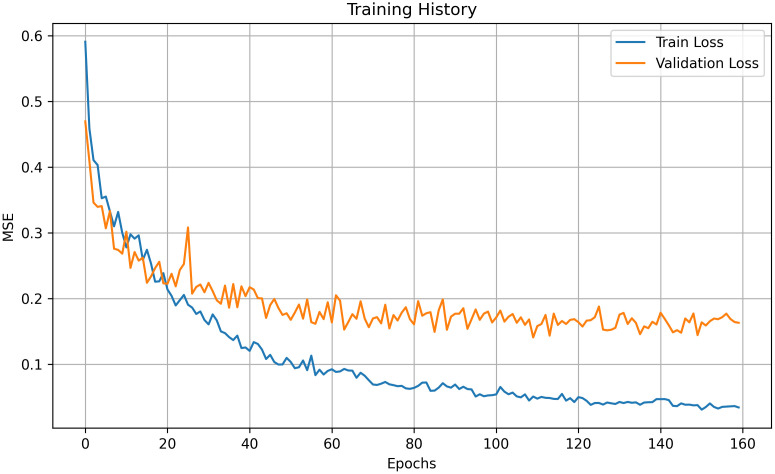
Training and validation loss curves of the Bi-LSTM model.

The curves for both the training and validation sets (Fig 4) show a rapid initial decrease in loss, followed by a slower stabilization. This pattern suggests that the model was progressively converging to an optimal solution. The use of early stopping (patience = 50) proved effective in preventing overfitting, as evidenced by the stable validation loss observed after the early epochs.

### Model validation

We compared weaning age estimates derived from our model (LSTM), WEAN, and ChangeR, using the Friedman test to assess overall differences. The individual isotopic profiles with all estimations displayed are shown in the Supplementary Material (S2–S21 Fig). The analysis yielded a test statistic of χ² = 3.111 with a p-value of 0.211, indicating no statistically significant differences among the three methods at the group level. Agreement between the methods was further examined using the CCC Showing that WEAN exhibits moderate agreement with our model (CCC = 0.532) and good agreement with ChangeR (CCC = 0.707). In contrast, the agreement between our model and ChangeR was weaker (CCC = 0.443). Bland-Altman plots among the three methods are included in the Supplementary Material (S22 Fig, S23 Fig, S24 Fig)

**Fig 5:**
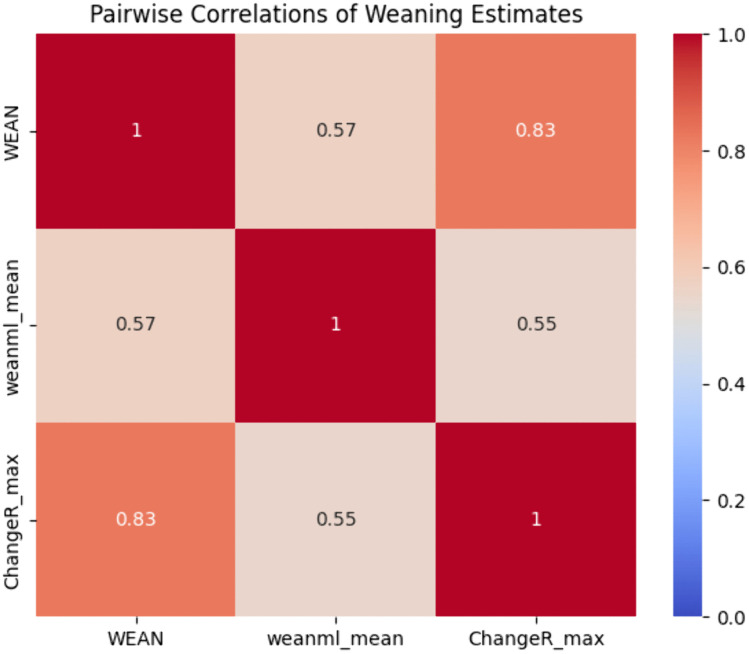
Heatmap showing pairwise correlations among the examined methods.

Pearson correlation coefficients echoed these findings (Fig 5), with WEAN correlating strongly with ChangeR (r = 0.827) and more moderately with our model (r = 0.570). The correlation between our model and ChangeR was comparatively low (r = 0.550), as shown in Fig 5.

**Fig 6:**
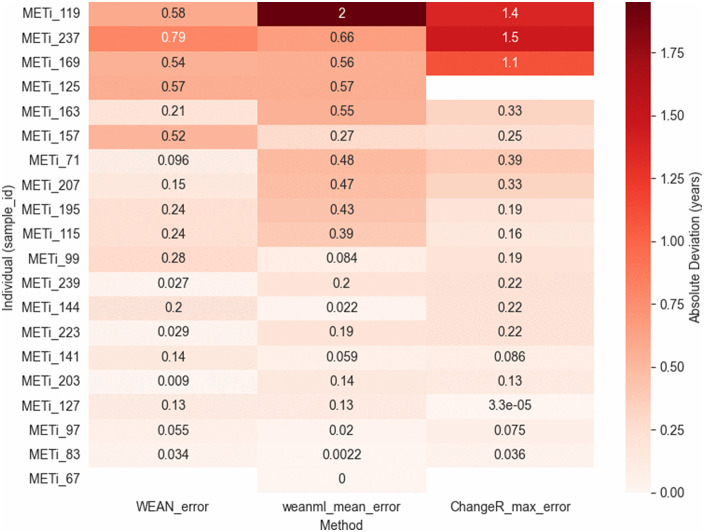
Heatmap showing the absolute prediction errors for the 20 Thessaloniki individuals among methods (WEAN, LSTM, ChangeR). Each row represents an individual and each column corresponds to a weaning estimation method. Redder colors indicate larger absolute errors.

Inspecting the estimates more closely, we identified three individuals (METi_119, METi_237, and METi_169) for whom the prediction error of our model exceeded 1 year. In all cases, our model disagreed most strongly with the ChangeR estimates, except for METi_119, where the largest discrepancy was with WEAN. These differences are summarized in Fig 6. Examining the individual plots, we can see that for METi_119, both WEAN and ChangeR estimate weaning between approximately 4 and 5 years, whereas our model predicts it before age 2.0, which appears more biologically plausible. For METi_237, ChangeR suggests a broad range between 2.5 and 4.5 years, while our model predicts 2.3–2.8 years, consistent with WEAN and overlapping with the lower portion of ChangeR’s range. A similar pattern is observed for METi_169, where our model’s estimate aligns more closely with WEAN than with ChangeR.

## Discussion

Our model demonstrates a novel and effective approach for estimating weaning duration, yielding an average prediction error of approximately 4.5 months. This is among the most precise and transparently validated computational estimation of weaning duration currently available, as no previous methods report prediction errors or confidence intervals.

This is the first model that captures the temporal dynamics of both δ^15^N and δ^13^C trajectories, accommodating both the temporal structure and the dietary changes, which enable a more robust handling of biological variability and observational noise than conventional statistical methods. In addition, Monte Carlo Dropout provides a means to quantify prediction uncertainty by generating confidence intervals around weaning age estimates, allowing the expression of the weaning duration as a range, rather than a single age. This is an important feature that reflects the gradual biological nature of weaning more realistically.

Among the three tooth types examined, the permanent first molar (M1) has the smallest interquartile range and appears most tightly distributed around zero. This strong performance suggests that δ^15^N and δ¹^3^C values preserved in the M1 may provide a more reliable temporal signal of dietary transitions during infancy. The M1 forms during the first days of life but later compared to deciduous teeth, which form in utero. This later development is possibly less influenced by changes in maternal diet during the last phases of pregnancy. As such, it is possible that this tooth type captures a broader or more stable dietary window during or after the weaning process and is more reliable to trace cumulative changes in nitrogen and carbon isotopic composition associated with the gradual introduction of non-maternal foods. The results of this study corroborate the results of previous studies that have identified the first permanent molar (M1) as the most suitable tooth within the permanent dentition for reconstructing weaning practices [17,19].

In contrast, the deciduous first molar (dM1) shows the largest spread with residuals extending from about −0.6 to +0.7 years and the second deciduous molar has an intermediate spread with a slight negative skew. Given that dM1 and dM2 begin formation in utero, the isotopic composition may be influenced by maternal dietary intake during late gestation, thereby introducing additional complexity and noise that confound clear weaning signals. Moreover, the early formation timing of these deciduous teeth may capture a narrower or less distinct dietary transition period. Apart from biological factors, methodological issues may hinder the predictive accuracy of these tooth types. Most importantly, both types are represented by substantially fewer measurements compared to M1, which may have limited the model’s ability to learn robust temporal patterns. Moreover, it is possible that parallel sectioning of dentine sampling may be more critical for these tooth types, an issue that has been previously raised by Tsutaya [19].

While deciduous teeth form early in life (S25 Fig) and their initial sections largely reflect in utero dietary intake, they still provide valuable insights into weaning. The later-formed segments of these teeth capture the critical transition from exclusive breastfeeding to complementary feeding, allowing the model to detect key isotopic changes. An advantage of using deciduous teeth is that they represent the entire early-life nutritional trajectory, including both prenatal and early postnatal periods, which can help contextualize weaning within broader physiological and developmental patterns. Moreover, deciduous teeth are often more numerous and better preserved in archaeological collections than permanent molars, expanding the available dataset and enabling model training across a wider range of individuals. By combining early and late segments in a sequence-aware framework, our model leverages these data while mitigating potential noise from in utero signals.

Nevertheless, future research could address these limitations by increasing sample sizes and improving temporal resolution through better sampling strategies. As additional data are incorporated into future iterations of the model, the role of dM1 and dM2 in reconstructing weaning practices warrants further investigation and their potential may not yet be fully realized. Our findings thus offer a valuable foundation for continued investigation into the developmental and dietary insights preserved in deciduous dentition. It is important to acknowledge that methodological procedures vary substantially between studies, for instance, in the sectioning protocols used or in the way age-at-increment is assigned. In our dataset alone, more than three different sectioning protocols were employed [14,26–32], and age-at-increment assignment followed anatomical, mathematical, or combined approaches [14,15,27–34]. While this heterogeneity inevitably introduces variability into our results, it also enhances the overall robustness and generalisability of our findings. Moreover, although our model uses time-ordered sequences, the underlying isotopic records are irregular. Some individuals have dense sampling, others sparse or uneven intervals. By padding and masking sequences, our model can ingest all available data while ignoring absent time points. Calculating differences, rolling averages, and slopes further normalized individuals to comparable temporal dynamics. This approach minimized bias introduced by sampling variation, but future work could test explicit imputation or irregular-time architectures (e.g., time-aware LSTMs) to fully exploit uneven sampling.

Furthermore, our dataset spans individuals from multiple centuries, encompassing diverse cultural and social contexts. Factors such as allomaternal care, female labour participation, and broader community practices likely influenced breastfeeding and weaning behaviours over time. A qualitative observation made during data compilation and testing, specifically among individuals later excluded from the final analysis, suggests that prehistoric samples display markedly different isotopic patterns compared to historic ones. This indicates that cultural and temporal variability may exert a measurable influence on our model’s predictions. As larger and more diverse datasets become available, it may become possible to develop more tailored or stratified models capable of distinguishing prehistoric from historic populations, thereby improving the resolution and cultural relevance of infant feeding reconstructions.

The validation of our LSTM model against previously established weaning estimation methods (WEAN and ChangeR) provides important insights into both performance and innovation. Although all three approaches yield broadly comparable weaning age estimates overall (non-significant Friedman test), WEAN and ChangeR demonstrate the strongest consistency with each other, whereas our model shows weaker alignment with both. This divergence likely reflects a fundamental methodological difference: unlike WEAN and ChangeR, which are rooted in predefined statistical rules, our model learns directly from the full isotopic time series. In other words, rather than simply reproducing a weaning estimate based on statistical computations, the model identifies complex, non-linear patterns embedded in the sequences.

A second key innovation is the incorporation of carbon isotopic ratios alongside nitrogen. While nitrogen has traditionally been the more robust indicator of breastmilk consumption, ongoing debates highlight the confounding effects of physiological stress and dietary supplementation. Many scholars argue that a truly unambiguous signal of weaning requires a parallel decline in both nitrogen and carbon isotopic values [35,36]. Our model operationalizes this concept by jointly calculating both Δ values and slopes across elements, thereby integrating two complementary isotopic dimensions into a single learning framework. This represents, to our knowledge, the first machine-learning approach to explicitly incorporate carbon as a co-determinant of weaning duration.

Finally, the fact that our model produces distinct estimates despite being trained on WEAN-based reference ages indicates that the model is not simply mimicking WEAN outputs but instead generalizing learned relationships from the underlying nitrogen and carbon patterns. This ability to extrapolate beyond the training heuristic is a critical step toward a more biologically grounded, data-driven understanding of weaning trajectories, particularly in cases of abrupt or atypical dietary transitions where traditional models may struggle.

Overall, the present results highlight the potential of LSTM models to integrate temporally structured isotopic data into meaningful reconstructions of infant feeding practices. At the same time, they emphasize the need for continued refinement in model architecture, the caution in model selection, the need for augmented training datasets, and a deeper understanding of tooth-specific developmental and dietary influences to fully exploit the capabilities of machine learning in bioarchaeological research.

## Conclusions

This study presents a novel application of a sequence-based Long Short-Term Memory (LSTM) model for estimating weaning duration from serial δ^15^N and δ^13^C measurements in human dentition. By jointly modelling nitrogen and carbon isotopic trajectories and incorporating temporal derivatives, rolling averages, and tooth-type encoding, our approach captures complex, non-linear dynamics in weaning signals that traditional statistical models cannot fully represent. The model achieved high predictive accuracy (R^2^ = 0.82; RMSE = 0.46 years) and robust performance across diverse samples, with the permanent first molar (M1) emerging as the most reliable indicator of weaning duration. These results corroborate previous findings on the suitability of M1 for reconstructing infant feeding practices, while also highlighting the greater variability and methodological challenges associated with deciduous teeth (dM1, dM2). Beyond predictive performance, this study demonstrates the value of integrating uncertainty estimation through Monte Carlo dropout, enabling confidence intervals around individual weaning predictions. This provides a more transparent and biologically realistic framework by acknowledging the inherent variability of isotopic data and the gradual nature of the weaning process.

Overall, the proposed model establishes a reproducible and scalable foundation for data-driven weaning reconstructions, offering new opportunities to integrate isotopic, developmental, and cultural data in bioarchaeological research. Future work with larger, temporally and geographically diverse datasets may further enhance model generalizability and enable refined interpretations of infant feeding behaviors across past populations.

## Supporting information

S1 Fig Distribution of paired δ^15^N and δ^13^C values per individual segregated by tooth type (M1, dM1, dM2). This figure shows the distribution of paired nitrogen (δ^15^N) and carbon (δ^¹³^C) isotopic measurements for each individual, separated by tooth type. The values illustrate variability related to tooth formation timing and dietary transitions.
(TIF)

S1 Table Dataset used to train the algorithm with annotated sample IDs, raw isotopic measurements (δ^15^N and δ^13^C), age-at-increment assignments, chronological dating and location information. The table contains annotated sample IDs, raw isotopic measurements (δ^15^N and δ^¹³^C), age-at-increment assignments, chronological dating, and location information used to train the weaning estimation algorithm.
(XLSX)

S2 Fig Individual isotopic profile of METi_83 showing paired measurements of δ^15^N and δ^13^C with overlayed weaning duration estimations according to WEAN, LSTM and ChangeR models. Paired δ^15^N and δ^13^C measurements per developmental increment with overlaid weaning duration estimations from the WEAN, LSTM, and ChangeR models.
(TIF)

S3 Fig Individual isotopic profile of METi_97 showing paired measurements of δ^15^N and δ^13^C with overlayed weaning duration estimations according to WEAN, LSTM and ChangeR models. Paired δ^15^N and δ^13^C measurements per developmental increment with overlaid weaning duration estimations from the WEAN, LSTM, and ChangeR models.
(TIF)

S4 Fig Individual isotopic profile of METi_99 showing paired measurements of δ^15^N and δ^13^C with overlayed weaning duration estimations according to WEAN, LSTM and ChangeR models. Paired δ^15^N and δ^13^C measurements per developmental increment with overlaid weaning duration estimations from the WEAN, LSTM, and ChangeR models.
(TIF)

S5 Fig Individual isotopic profile of METi_67 showing paired measurements of δ^15^N and δ^13^C with overlayed weaning duration estimations according to WEAN, LSTM and ChangeR models. Paired δ^15^N and δ^13^C measurements per developmental increment with overlaid weaning duration estimations from the WEAN, LSTM, and ChangeR models.
(TIF)

S6 Fig Individual isotopic profile of METi_71 showing paired measurements of δ^15^N and δ^13^C with overlayed weaning duration estimations according to WEAN, LSTM and ChangeR models. Paired δ^15^N and δ^13^C measurements per developmental increment with overlaid weaning duration estimations from the WEAN, LSTM, and ChangeR models.
(TIF)

S7 Fig Individual isotopic profile of METi_203 showing paired measurements of δ^15^N and δ^13^C with overlayed weaning duration estimations according to WEAN, LSTM and ChangeR models. Paired δ^15^N and δ^13^C measurements per developmental increment with overlaid weaning duration estimations from the WEAN, LSTM, and ChangeR models.
(TIF)

S8 Fig Individual isotopic profile of METi_207 showing paired measurements of δ^15^N and δ^13^C with overlayed weaning duration estimations according to WEAN, LSTM and ChangeR models. Paired δ^15^N and δ^13^C measurements per developmental increment with overlaid weaning duration estimations from the WEAN, LSTM, and ChangeR models.
(TIF)

S9 Fig Individual isotopic profile of METi_223 showing paired measurements of δ^15^N and δ^13^C with overlayed weaning duration estimations according to WEAN, LSTM and ChangeR models. Paired δ^15^N and δ^13^C measurements per developmental increment with overlaid weaning duration estimations from the WEAN, LSTM, and ChangeR models.
(TIF)

S10 Fig Individual isotopic profile of METi_237 showing paired measurements of δ^15^N and δ^13^C with overlayed weaning duration estimations according to WEAN, LSTM and ChangeR models. Paired δ^15^N and δ^13^C measurements per developmental increment with overlaid weaning duration estimations from the WEAN, LSTM, and ChangeR models.
(TIF)

S11 Fig Individual isotopic profile of METi_239 showing paired measurements of δ^15^N and δ^13^C with overlayed weaning duration estimations according to WEAN, LSTM and ChangeR models. Paired δ^15^N and δ^13^C measurements per developmental increment with overlaid weaning duration estimations from the WEAN, LSTM, and ChangeR models.
(TIF)

S12 Fig Individual isotopic profile of METi_141 showing paired measurements of δ^15^N and δ^13^C with overlayed weaning duration estimations according to WEAN, LSTM and ChangeR models. Paired δ^15^N and δ^13^C measurements per developmental increment with overlaid weaning duration estimations from the WEAN, LSTM, and ChangeR models.
(TIF)

S13 Fig Individual isotopic profile of METi_144 showing paired measurements of δ^15^N and δ^13^C with overlayed weaning duration estimations according to WEAN, LSTM and ChangeR models. Paired δ^15^N and δ^13^C measurements per developmental increment with overlaid weaning duration estimations from the WEAN, LSTM, and ChangeR models.
(TIF)

S14 Fig Individual isotopic profile of METi_157 showing paired measurements of δ^15^N and δ^13^C with overlayed weaning duration estimations according to WEAN, LSTM and ChangeR models. Paired δ^15^N and δ^13^C measurements per developmental increment with overlaid weaning duration estimations from the WEAN, LSTM, and ChangeR models.
(TIF)

S15 Fig Individual isotopic profile of METi_163 showing paired measurements of δ^15^N and δ^13^C with overlayed weaning duration estimations according to WEAN, LSTM and ChangeR models. Paired δ^15^N and δ^13^C measurements per developmental increment with overlaid weaning duration estimations from the WEAN, LSTM, and ChangeR models.
(TIF)

S16 Fig Individual isotopic profile of METi_169 showing paired measurements of δ^15^N and δ^13^C with overlayed weaning duration estimations according to WEAN, LSTM and ChangeR models. Paired δ^15^N and δ^13^C measurements per developmental increment with overlaid weaning duration estimations from the WEAN, LSTM, and ChangeR models.
(TIF)

S17 Fig Individual isotopic profile of METi_195 showing paired measurements of δ^15^N and δ^13^C with overlayed weaning duration estimations according to WEAN, LSTM and ChangeR models. Paired δ^15^N and δ^13^C measurements per developmental increment with overlaid weaning duration estimations from the WEAN, LSTM, and ChangeR models.
(TIF)

S18 Fig Individual isotopic profile of METi_125 showing paired measurements of δ^15^N and δ^13^C with overlayed weaning duration estimations according to WEAN, LSTM and ChangeR models. Paired δ^15^N and δ^13^C measurements per developmental increment with overlaid weaning duration estimations from the WEAN, LSTM, and ChangeR models.
(TIF)

S19 Fig Individual isotopic profile of METi_127 showing paired measurements of δ^15^N and δ^13^C with overlayed weaning duration estimations according to WEAN, LSTM and ChangeR models. Paired δ^15^N and δ^13^C measurements per developmental increment with overlaid weaning duration estimations from the WEAN, LSTM, and ChangeR models.
(TIF)

S20 Fig Individual isotopic profile of METi_115 showing paired measurements of δ^15^N and δ^13^C with overlayed weaning duration estimations according to WEAN, LSTM and ChangeR models. Paired δ^15^N and δ^13^C measurements per developmental increment with overlaid weaning duration estimations from the WEAN, LSTM, and ChangeR models.
(TIF)

S21 Fig Individual isotopic profile of METi_119 showing paired measurements of δ^15^N and δ^13^C with overlayed weaning duration estimations according to WEAN, LSTMand ChangeR models. Paired δ^15^N and δ^13^C measurements per developmental increment with overlaid weaning duration estimations from the WEAN, LSTM, and ChangeR models.
(TIF)

S22 Fig Bland-Altman plot comparing LSTM (this study) and ChangeR estimates. Shows agreement and bias between weaning age predictions from LSTM and ChangeR models.
(TIF)

S23 Fig Bland-Altman plot comparing WEAN and ChangeR estimates. Shows agreement and bias between weaning age predictions from WEAN and ChangeR models.
(TIF)

S24 Fig Bland-Altman plot comparing TLSM (this study) and WEAN estimates. Shows agreement and bias between weaning age predictions from LSTM and WEAN models.
(TIF)

S25 Fig Schematic illustration of deciduous and permanent developmental timings. **Timings were approximated following the London Atlas** Development timelines were approximated following the London Atlas standards. The figure illustrates crown formation, initiation, and completion stages for deciduous and permanent teeth. Adapted from [37].
(TIF)
